# Polystyrene Nanoplastics Can Alter the Toxicological Effects of Simvastatin on *Danio rerio*

**DOI:** 10.3390/toxics9030044

**Published:** 2021-02-26

**Authors:** Angela Barreto, Joana Santos, Mónica J.B. Amorim, Vera L. Maria

**Affiliations:** Department of Biology & CESAM, University of Aveiro, 3810-193 Aveiro, Portugal; abarreto@ua.pt (A.B.); joanasilvasantos@ua.pt (J.S.); mjamorim@ua.pt (M.J.B.A.)

**Keywords:** plastics, pharmaceuticals, ecotoxicology, aquatic organisms, co-exposure

## Abstract

Once in the environment, nanoplastics (NPls) may interact with other contaminants, such as pharmaceuticals, potentially acting as carriers and modulating their toxicity. Thus, the main aim of the current study is to investigate how polystyrene (PS) NPls (mean diameter: 60 nm) interact with simvastatin (SIM), an anticholesterolemic drug, and modulate its toxicity to zebrafish (*Danio rerio*) embryos. PS NPls were carboxyl group functionalized, to promote the interaction/binding of NPls with SIM (worst-case scenarios) and it was fluorescently dyed, allowing to detect the intake. Exposure was 96 h to 0–150 mg/L NPls or 0–150 µg/L SIM, as well as to dual combinations (NPls 0.015 or 1.5 mg/L and SIM 12.5 or 15 µg/L). PS NPls alone did not exert effects whereas SIM (≥12.5 µg/L) significantly delayed the hatching, decreased the heartbeat, induced edemas and mortality. The combination of NPls (1.5 mg/L) and SIM (12.5 or 15 µg/L) had significant effects on the survival of the organisms while the correspondent NPls and SIM single exposures did not have significant effects on this endpoint. Concerning the malformations appearance, SIM alone had similar effects than when in co-exposures (0.015 mg/L NPls plus 12.5 or 15 µg/L SIM). Hatching and heartbeat increased after the co-exposures SIM and NPls comparing with SIM single exposures, showing that 0.015 mg/L NPls plus 12.5 or 15 µg/L SIM did not cause significant effects on these endpoints. This study shows that NPls effects on bioavailability and toxicity of other contaminants cannot be ignored when assessing the environmental behavior and risks of NPls.

## 1. Introduction

Plastic particles are produced and released to the environment from industrial use, human activities and inadequate waste management [1,2]. Despite the definition of nanoplastics (NPls) to be controversial, the most accepted one defines NPls as particles unintentionally produced (i.e., from the degradation and the manufacturing of the plastic objects) and presenting a colloidal behavior, within a size range from 1 to 1000 nm [3,4,5]. Due to limitations in analytical methods, the quantification of NPls in the environment remains a challenge [6,7,8]. However, it is foreseeable that the levels of NPls in the environment (in specific aquatic systems) are increasing consistently over time due to the continuous release and consequent degradation of macro-/microplastics [4,9,10,11]. Once in the environment, NPls exhibit chemical and physical characteristics different from those of bulk plastics. Therefore, their environmental fate, bioavailability, intake and potential impact to the organisms should be investigated [11]. Some authors reported that NPls are able to cross the biological barriers and accumulate in tissues of aquatic organisms like adult and embryos of fish medaka (*Oryzias latipes*) and embryos of zebrafish (*Danio rerio*) [5,12]. This NPls bioaccumulation will depend, among other factors, on the NPls’ size (chorion pore size of zebrafish is 600–700 nm) [4]. Despite the increasing number of studies regarding the toxicity of NPls in aquatic organisms [4,5,9,10,13], the knowledge about their environmental risk, bioaccumulation and the mechanisms involved in their toxicity is yet limited [3,4,5]. On zebrafish embryos, polystyrene (PS) NPls showed to be able to penetrate the chorion, accumulate on the embryos and induce bradycardia and hypoactivity [10,13] Table 1. However, no significant effect on survival, hatching and embryos morphology was found [3,4,11,14,15].

Another important aspect is that, due to their hydrophobicity and large surface area, NPls are capable, physically or chemically, to adsorb onto other contaminants present in the environment [5,8,15]. As plastic particles have been observed to be ingested by aquatic organisms, there is an increasing concern about their “vector” role for other contaminants [1]. Considering that, aquatic organisms, e.g., fish, might be exposed to a mixture of contaminants, the comprehension of the potential role of the NPls in the bioavailability and in the biological effects of other contaminants is highly needed. Combined exposures of NPls and other contaminants have shown differential toxicity to zebrafish [1,3,15]. Zebrafish is widely used as a model species in toxicity testing [16,17] and it was already used to investigate NPls ability as a vector to Au ions, polycyclic aromatic hydrocarbons (PAHs), bisphenol A and 17 α-ethynylestradiol [1,3,5,15].

Pharmaceuticals are considered contaminants of emerging concern due to, among other reasons: (1) their high consumption and continuous environmental release (as parental compound, metabolites and/or transformation products); (2) inefficient wastewater treatment processes; (3) high environmental persistence; (4) low degradation rates [18,19]. Albeit pharmaceuticals are found in trace amounts in aquatic environment, they were designed to produce a biological effect to target organisms at low concentrations [20,21]. In fact, several pharmaceuticals have been demonstrated to induce adverse effects at environmentally relevant concentrations to non-target organisms [16]. Simvastatin (SIM), among the most prescribed pharmaceuticals in western countries, is a hypolipidemic drug belonging to the statin class and used as the primary treatment of hypercholesterolemia [17,22,23]. With the increasing SIM consumption and subsequently discharge into the aquatic environment, this pharmaceutical has been detected at low concentrations ranging from 0.1 to 1560 ng/L [17,24,25]. It has been reported that SIM presents a high environmental persistence, a high bioaccumulation and induces adverse effects to non-target aquatic organisms at environmentally relevant concentrations [23,25,26,27]. Accordingly, SIM caused several effects to zebrafish embryos such as morphological damage (e.g., pericardial and yolk sac edemas), delayed hatching, reduced heart beating and impaired larvae swimming capacity [16,24,26,28].

Overall, the present research hypothesizes that PS NPls alter the toxic effects of SIM to the *D. rerio* embryos, being expected antagonistic and synergistic effects. Thus, the main aim of this study is to assess how functionalized PS NPls interact with SIM and modulate its toxicity to zebrafish embryos. The survival, heartbeat, hatching and morphology of embryos/larvae will be analyzed during 96 h of exposure.

## 2. Materials and Methods

### 2.1. Test Organism

Zebrafish (*D. rerio*) wild type AB eggs were obtained from a culture maintained at the Department of Biology, University of Aveiro (Aveiro, Portugal). Zebrafish adults were kept in a recirculating system with reverse osmosis and activated carbon filtered tap water, complemented with instant ocean synthetic salt automatically adjusted for pH and conductivity. The organisms were maintained at 26.0 ± 1°C, under a 16:8 h light/ dark photoperiod cycle, with conductivity at 750 ± 50 µS/cm, pH at 7.5 ± 0.5, salinity of 0.35 and dissolved oxygen at 95% saturation. Adult fishes were fed daily with commercially artificial diet, Gemma Micro 500 (Skretting^®^, Burgos, Spain). Reproduction groups of zebrafish adults were placed in aquarium with marbles in the bottom, in the afternoon of the day before the collection of the eggs. Two hours after the beginning of the illumination in the next morning, the eggs were collected and cleaned from residues. Zebrafish eggs with normal development were selected for the toxicity test, using a SMZ 1500 Stereoscopic Zoom Microscope (Nikon, Amsterdam, The Netherlands). Unfertilized, irregular or injured eggs were discarded.

### 2.2. Preparation and Characterization of Nanoplastics

Functionalized (with carboxyl group -COOH) PS NPls (catalog code FC02F; mean nominal diameter: 60 nm) were acquired from Bangs Laboratories, Inc. (Immunostep, Salamanca, Spain). Functionalized PS NPls were selected to potentiate/promote the interaction/binding of NPls with SIM. PS NPls are also fluorescent dyed by dragon green with excitation/emission wavelength (nm) of 480/520, respectively. According to the manufacturer, this dye is internal linked and stable within the bead under most aqueous conditions (including 0.01% methanol used for the exposure conditions with SIM in the fish bioassays). In addition, the stock dispersion had 1% of NPls, 0.1% tween 20 and 2 mM sodium azide. The Supplementary Table S1 presents some characteristics of the PS NPls stock dispersion used. Prior to the bioassays, the particles were centrifuged using a Vivaspin^®^ 2 mL ultrafiltration device (Bangs Laboratories, Inc.) to remove the sodium azide and tween 20 present in the NPls stock dispersion. The procedure was made using the manufacturer indication included in the Vivaspin^®^ device.

The NPls dispersion resultant from the centrifugation was characterized by hydrodynamic size, assessed by dynamic light scattering (DLS; Zetasizer Nano ZS, Malvern, USA); by primary size and shape, evaluated by the transmission electron microscopy (TEM; Hitachi, H9000 NAR, Tokyo, Japan) and scanning electron microscopy (SEM; Hitachi, SU70, Tokyo, Japan); and by zeta potential (ZP), assessed by electrophoretic light scattering (Zetasizer Nano ZS, Malvern). Characterization of the NPls—hydrodynamic size and ZP—was also performed in the medium used in the bioassays, at 0 and 96 h (corresponding to the beginning and to the end of the tests). To investigate the interaction between NPls and SIM (acquired from Acros Organics^TM^, Fisher Scientific, Darmstadt, Germany), hydrodynamic size and ZP were also assessed at the tested exposure combinations.

### 2.3. Fish Assays

The test solutions were prepared 24 h before the beginning of the experiments, adding the required volumes of the NPls and SIM in the medium used in the bioassays, followed by a brief stirring. The solutions were pre-incubated overnight prior to the experiment due to the presence of the contaminant mixture (NPls + SIM) as previously performed for the combination of NPls with other contaminants [5].

The assays were based on the OECD guideline on Fish Embryo Toxicity (FET) test [29]. Five embryos per glass petri dish (Diameter = 3.2 cm) were used (*n* = 4, 20 embryos per condition). Per embryo, 0.5 mL of medium was considered. Embryos were exposed to the single exposures: 0; 0.015; 1.5 and 150 mg/L of NPls or 0; 0.15; 1.5; 12.5; 15 and 150 µg/L of SIM and to the combinations: 0.015 mg/L of NPls + 12.5 µg/L of SIM; 0.015 mg/L of NPls + 15 µg/L of SIM; 1.5 mg/L of NPls + 12.5 µg/L of SIM and 1.5 mg/L of NPls + 15 µg/L of SIM. The lowest NPls tested concentration (15 µg/L) has been predicted to be environmentally relevant concentration for the aquatic environment [30]. The other tested concentrations were 100-fold increases. The lowest SIM tested concentrations (0.15 and 1.5 µg/L) are environmentally relevant concentrations [23]. The other tested concentrations were 10-fold increases (15 and 150 µg/L). In addition, an intermediate value (12.5 µg/L) was tested to ensure at least two concentrations of SIM inducing sub-lethal effects to zebrafish embryos (to be tested at combined exposures). The chosen concentrations for the combined exposures were based on the concentrations causing sub-lethal effects in the individual exposures. If no significant effect was detected, the lowest tested concentrations were selected due to their environmental relevance. Due to the low solubility of SIM in water, we also used a solvent control—methanol—at 0.01%, the concentration of methanol present in the treatments with SIM not exceeding the value recommended by the OECD guideline [31]. The test ran for 96 h, at 26 ± 1 °C, and embryos/larvae were observed daily with a stereomicroscope. Egg coagulation and larvae mortality, the presence of malformations (such as pericardial/yolk sac edemas and tail deformation), hatching and heartbeat were evaluated during embryogenesis.

### 2.4. Statistical Analysis

Graphics and statistical treatment were performed using the Sigma Plot 12.5 software package (Systat Software Inc., Munich, Germany). Shapiro-Wilk and Levene’s tests were done to assess the normality and homoscedasticity of data, respectively. Differences between control and solvent control were carried out using a Student t-test. If data passed the normality and homoscedasticity tests, one-way analysis of variance (ANOVA) followed by Dunnett’s multiple comparison post hoc test was used to assess differences between control and treatments. One-way ANOVA followed by Tukey test was also used to compare differences between single and combined exposures. When data failed the normality and/or homoscedasticity tests, a non-parametric Kruskal-Wallis’ test was performed. Significant differences were assumed for a significance level (*p*) < 0.05. Median effect concentrations (EC_50_) were estimated using the Toxicity Relationship Analysis Program (TRAP v1.22) (Washington, DC, USA) or the Sigma Plot 12.5 software.

## 3. Results

### 3.1. Characterization of Nanoplastics Alone and with Simvastatin

At 0 and 96 h, in the test media without organisms, NPls hydrodynamic size and ZP were similar than the ones measured in the stock dispersion (Table 2). After 96 h, both in the test media with organisms and with SIM, the NPls hydrodynamic size increased for all the tested concentrations (Table 2). In terms of NPls ZP, this value increased in the test media containing SIM (Table 2). The analysis by electron microscopy (TEM and SEM) showed that NPls are mostly spherical (Supplementary Figure S1) with an average diameter of 55 nm.

### 3.2. Effects of Single Exposures

#### 3.2.1. Effects of Nanoplastics

**Figure 1:**
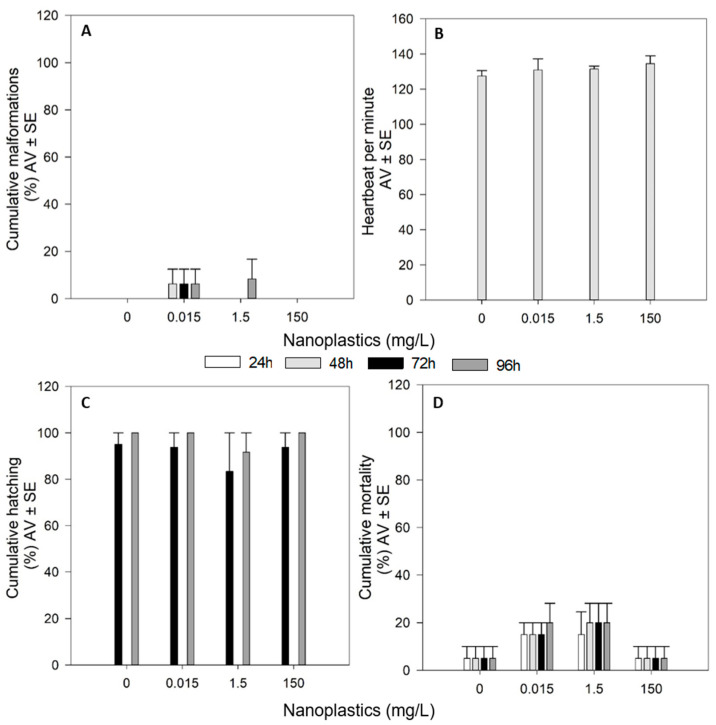
Effects of functionalized polystyrene nanoplastics on malformations appearance (**A**), heartbeat (**B**), hatching (**C**) and mortality (**D**) on zebrafish (*Danio rerio*) embryos/larvae exposed for 96 h. Results are expressed in average value (AV) ± standard error (SE).

NPls single exposures caused no significant effects on the survival and hatching rates of the organisms during the 96 h of the exposure (*p* > 0.05, Figure 1C,D). Additionally, no significant malformations were induced by NPls during the time of the exposure (*p* > 0.05, Figure 1A). The assessment of the heartbeat of the organisms, at 48 h, showed that NPls did not cause a significant effect on this endpoint (*p* > 0.05, Figure 1B).

#### 3.2.2. Effects of Simvastatin

**Figure 2:**
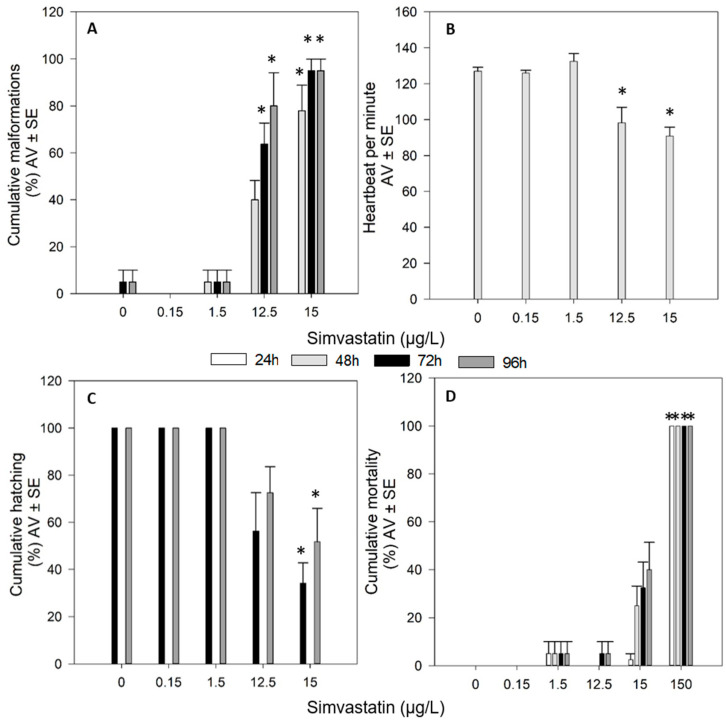
Effects of simvastatin on malformations appearance (**A**), heartbeat (**B**), hatching (**C**) and mortality (**D**) on zebrafish (*Danio rerio*) embryos/larvae exposed for 96 h. Results are expressed in average value (AV) ± standard error (SE). * Significant differences to control (*p* < 0.05).

In the bioassays testing the effects of SIM, there were no significant differences between the control and the solvent control (*p* > 0.05). Therefore, the differences were assessed between treatments and the control group. SIM, at 150 µg/L, caused 100% of mortality in less than 24 h of exposure (*p* < 0.05). Although not significant, 15 µg/L SIM produced approximately 40% of mortality (*p* > 0.05) for 96 h of exposure (Figure 2D). Additionally, 12.5 and 15 µg/L SIM significantly induced edemas and other malformations (pericardial edemas, yolk sac edemas and tail malformations) on the organisms (Figure 2A). The assessment of the heartbeat of the organisms at 48 h, showed that 12.5 and 15 µg/L SIM significantly induced a reduction of the number of heartbeats per minute (*p* < 0.05, Figure 2B). A significant delay in the hatching of the organisms was also detected at 15 µg/L SIM (*p* < 0.05, Figure 2C).

**Figure 3:**
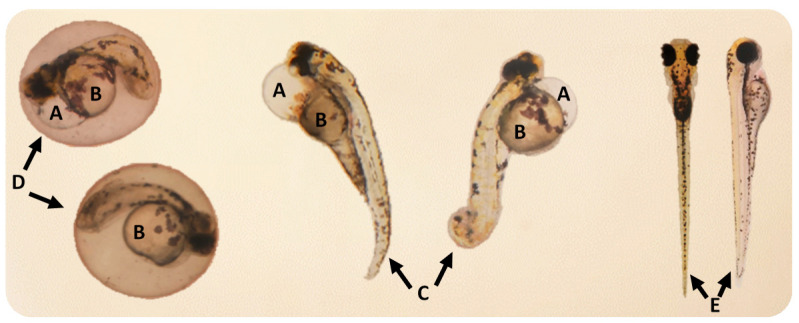
Malformations detected in the organisms after the exposure to 15 µg/L of simvastatin, from 48 up to 96 h exposure: (**A**) Pericardial edema; (**B**) Yolk sac edema; (**C**) Tail malformation; (**D**) Delayed hatching at 96 h of exposure; (**E**) Normal larvae.

From 48 up to 96 h exposure, the pericardial edemas, yolk sac edemas and tail malformations observed in the organisms after the exposure to 15 µg/L of SIM are presented in Figure 3.

### 3.3. Effects of Combined Exposures Nanoplastics and Simvastatin

**Figure 4:**
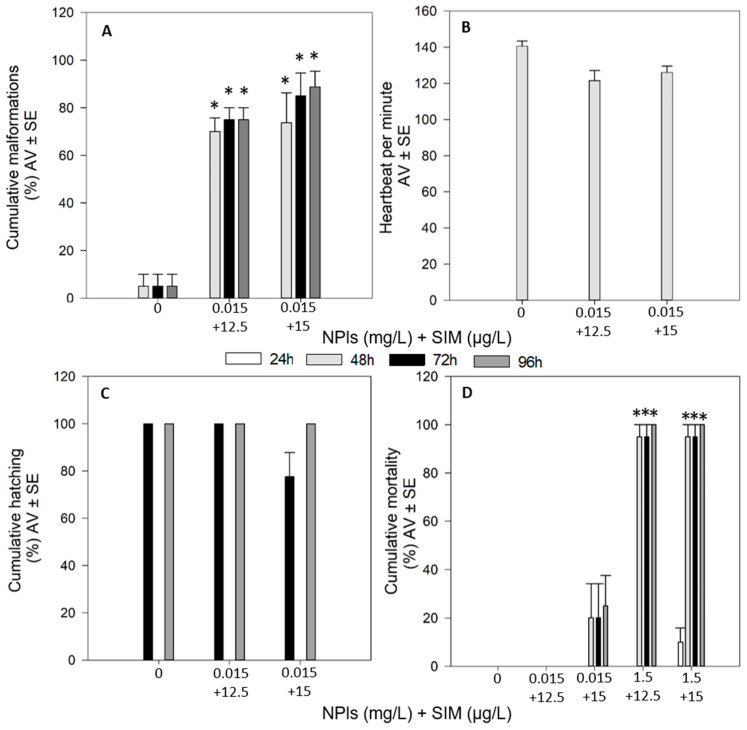
Effects of combined exposure of functionalized nanoplastics (NPls) with simvastatin (SIM) on malformations appearance (**A**), heartbeat (**B**), hatching (**C**) and mortality (**D**) on zebrafish (*Danio rerio*) embryos/larvae exposed for 96 h. Results are expressed in average value (AV) ± standard error (SE). * Significant differences to control (*p* < 0.05).

The combinations 1.5 mg/L NPls + 12.5 µg/L SIM and 1.5 mg/L NPls + 15 µg/L SIM caused mortality of all the organisms at less than 48 h (*p* < 0.05, Figure 4D). The other two combinations tested (0.015 mg/L NPls + 12.5 µg/L SIM and 0.015 mg/L NPls + 15 µg/L SIM) have no significant effects in terms of mortality and hatching percentages during the 96 h of exposure (*p* > 0.05, Figure 4C,D). Also, heartbeat of the organisms, assessed at 48 h, was not altered by 0.015 mg/L NPls + 12.5 µg/L SIM and 0.015 mg/L NPls + 15 µg/L SIM (*p* > 0.05, Figure 4B). However, these two combinations induced significant malformations appearance on the organisms, from 48 up to 96 h of exposure (*p* < 0.05, Figure 4A).

**Figure 5:**
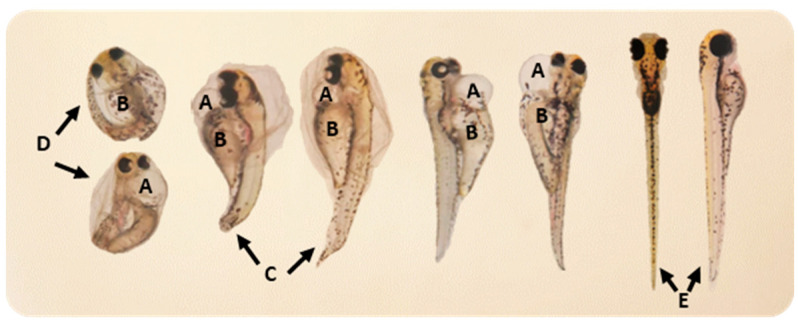
Malformations detected in the organisms after the exposure to combined exposures of nanoplastics (0.015 mg/L) and simvastatin (12.5 or 15 µg/L), from 48 up to 96 h exposure. (**A**) Pericardial edema; (**B**) Yolk sac edema; (**C**) Tail malformation; (**D**) Delayed hatching at 72 h of exposure detected on the combination 0.015 mg/L NPls + 15 µg/L SIM; (**E**) Normal larvae.

From 48 up to 96 h exposure, the malformations—pericardial edemas, yolk sac edemas and tail malformations—observed in the organisms caused by the exposure to the combinations: 0.015 mg/L NPls + 12.5 µg/L SIM and 0.015 mg/L NPls + 15 µg/L SIM are presented in Figure 5.

The EC_50_ for survival was around 15 µg/L in SIM single exposure and in combination with 0.015 mg/L NPls, whereas when SIM was in combination with 1.5 mg/L NPls the EC_50_ decreased to around 7 µg/L (Table 3). The EC_50_ for the appearance of malformations was similar when SIM was alone or combined with NPls. However, for hatching and heartbeat, the EC_50_ increased when SIM was in the presence of NPls (Table 3).

Comparing the effects of single versus combined exposures (Table 4), in terms of survival, the combinations 1.5 mg/L NPls with 12.5 or 15 µg/L SIM induced 100% mortality (*p* < 0.05), whereas the single exposures did not cause any significant effect (comparing with the control group (*p* > 0.05)). In relation to hatching and heartbeat assessment, the combinations 0.015 mg/L NPls with 12.5 or 15 µg/L SIM caused no significant effects on these endpoints (*p* > 0.05). However, the single exposures of SIM (both 12.5 and 15 µg/L) caused a significant decrease on the heartbeat and 15 µg/L caused a significant decrease on the hatching of the organisms (*p* < 0.05). The occurrence of malformations after the exposure to SIM was independent of the presence or absence of NPls: SIM combined with 0.015 mg/L NPls or SIM in single exposures induced significant malformations on the exposed organisms (*p* < 0.05).

## 4. Discussion

NPls maintained their hydrodynamic size during the time of the exposure test (96 h) in the medium used for the bioassays. Moreover, no alterations in terms of ZP were found between 0 and 96 h, showing the stability of the tested NPls even when in the test medium, as previously reported [5]. However, the NPls’ hydrodynamic size and ZP increased in the presence of SIM in the medium used for the fish bioassays. Moreover, the characterization of NPls on the test medium with the organisms, showed an increase of their hydrodynamic size during the 96 h of exposure, which can be related with the interaction of NPls with embryos/larvae metabolites present in the test media [32,33]. Despite the hydrodynamic size enlargement of NPls, to around 300 nm, it does not seem to limit their entrance (through the chorion of zebrafish embryos), since the pore size of this structure is between 600 to 700 nm [4].

To our knowledge, in most of the studies assessing the effects of NPls with other contaminants on zebrafish, the NPls’ characterization was only performed when they were present alone and it is not referred if the characterization is performed in the presence or absence of organisms [1,3,5]. Only one study reported a fast aggregation of NPls (increased sizes) when they were in the presence of PAHs [16]. The authors reported that the sorption of PAHs to the surface of NPls is expected to decrease the interaction with cations available in the exposure medium and to increase the lipophilicity of NPls, promoting further aggregation and sorption of PAHs [15]. An identical interaction is likely to have occurred on our study in the presence of the pharmaceutical SIM. Indeed, PS NPls were functionalized with COOH, which can promote the interaction/binding of NPls with SIM. Additionally, the characterization of NPls with SIM showed that, behind the increase in the NPls hydrodynamic size, the surface charge of the NPls also increased (ZP from −25 to −12 mV), which may be due to the interaction/binding of NPls with SIM.

The interaction of NPls with SIM and consequent alteration of the NPls’ chemical characteristics may induce different biological effects comparing with the effects when NPls are alone. Moreover, SIM chemical properties may also be altered in the presence of NPls promoting differential effects compared to individual SIM exposure. The results showed no effects induced by PS NPls, indicating that NPls, at the tested concentrations, did not cause a significant acute toxic effect on the embryonic development of zebrafish. However, the pharmaceutical SIM presented a dose dependent toxicity, causing mortality, malformations, hatching and heartbeat impairment in the organisms. Lee et al. [5] also showed no significant effects of PS NPls (50, 200 and 500 nm) on zebrafish embryogenesis. For SIM, Ribeiro et al. [16] also reported toxic effects on zebrafish embryos development, at the endpoints assessed in our study.

The adsorption (physical or chemical) of diverse compounds [5,15] to NPls is widely known, and hence the high relevance of investigating the combined toxic effect of NPls and SIM. To the best of our knowledge, until now, few studies have focused on the effects of NPls on the toxicity of other contaminants on zebrafish [1,3,5,15]. Further, the combined exposures of NPls with other contaminants have shown diverse outcomes regarding toxicity [1,3,5,15]. It is thus important to comprehend in further detail how plastics can interact with other contaminants and modulate their uptake and further toxicity. Such effects can be difficult to predict, as they are dependent on the polymer type, size and charge of the plastic particles, type of co-contaminant and their concentrations [15]. In our study, the highest NPls concentration tested in the combined exposures (1.5 mg/L) appears to work as an effective and a greater carrier of SIM (as opposed to 0.015 mg/L NPls) leading to fast (<24 h) lethal effects on the zebrafish embryos. Decreasing the NPls concentrations, the lethal effect disappeared although sub-lethal effects persisted. Concerning the mortality, the combination of NPls and SIM had relevant effects on the survival of the organisms whereas the single exposures did not have significant effects on this endpoint. A previous study already reported that NPls themselves may not be severely detrimental, but when they are combined with environmental contaminants, such as metal ions, the toxic effects may escalate [12]. The median lethal concentration (LC_50_) of Au ion without NPls was 1.88 μg/mL, whereas it was 1.25 μg/mL in the presence of NPls, indicating that the presence of NPls exacerbated the mortality effect of the Au ion on zebrafish embryos [5]. In the present study, a similar result was also found for survival, and the LC_50_ for the combination SIM + NPls was lower than for SIM alone, hence there is a synergy beyond simple additivity.

Concerning the malformations appearance, SIM single exposures had similar effects than when in co-exposures (EC_50_ were similar between SIM single and combined exposures), the SIM mode action seems independent on the presence of NPls (at 0.015 mg/L). These results are not similar to the combination of NPls with Au ion since the Au ion induction malformations was synergistic when combined with NPls [5]. Contrary to Lee et al. [5] and to our work, PAHs single exposures caused higher rates of developmental deformities, but the co-exposure of PAHs with NPls showed a decreased effect [15], hence antagonistic combination. In terms of hatching and heartbeat, a protective effect was found in our study after the co-exposures SIM and NPls comparing with SIM single exposures. It seems that the presence of NPls, at 0.015 mg/L, alleviated the effects of SIM on these endpoints, i.e., EC_50_ for the combination of SIM and NPls was higher than for SIM alone. However, other authors have reported that NPls synergistically accelerated the inhibition of hatching caused by the Au ion [5]. The presence of the lower concentration of NPls in the combined exposures seems to offer a protective shield to embryos. The possible absorption of SIM to NPls may result in less bioavailability of the pharmaceutical to the organisms and consequent less interaction, resulting in sub-lethal effects (hatching and heartbeat) less pronounced compared with the effects resulting from the SIM single exposures. A previous study also showed that NPls co-exposure with bisphenol A alleviated the effects of bisphenol A on the AChE activity: combined exposure did not inhibit the AChE activity of zebrafish, whereas bisphenol A single exposure caused an inhibition [1].

Our hypothesis was confirmed by the obtained results, for some endpoints (hatching and heartbeat) antagonistic effects were found, i.e., the combined effect of NPls and SIM was less toxic than the individual effects. Whereas for other endpoints (survival), synergistic effects were detected, i.e., the combined effect of NPls and SIM was much greater than the sum of the effects of each contaminant alone. However, for malformations appearance, additive effects occurred, i.e., the combined effect of NPls and SIM was similar to the sum of the effect of each contaminant alone. It seems that the biological processes are timely expressed, i.e., at the combined exposures, the adverse effects were earlier revealed (i.e., mortality at <24 h for 1.5 mg/L NPls + 12.5 or 15 µg/L SIM and malformations development in <48 h for 0.015 mg/L NPls + 12.5 µg/L SIM), when compared with the correspondent SIM single exposures. However, the survived embryos showed deal better with the “hostile environment” (combined exposures), occurring a normal hatching process (at 72 h) and heartbeat (48 h), on contrary to the embryos in the SIM single exposures.

Overall, the concentration of NPls and the chemical properties of the co-contaminant seem to be relevant for further toxicological effects on organisms. The mechanisms behind the altered toxicity of SIM by the presence of NPls should be more explored, but may be associated with incorporation rates, sorbing ability, cellular defense mechanisms and different modes of action. NPls are capable of physically or chemically adsorb to biomolecules in living organisms, such as proteins, lipids, and metabolites in serum and cytoplasm [32,33]. This adsorption ability results from the hydrophobic surface and large surface area of NPls and may alter the dynamics of the surface ionic charges in the environmental context [34,35] and, eventually, resulting in altered toxicity of the complex containing NPls and adsorbed elements [5]. Moreover, the effects of NPls on the toxicity of other contaminants can also be dependent on the NPls concentration (it seems our case) and/or of the type of the compounds as previously reported by other authors [3,5]. Although we were not able to perform an absolute/relative quantification of the NPls uptake, it may be possible to observe in the photos (provided as Supplementary Information, Supplementary Figure S2) an increase on the intensity of green fluorescence with the increase of NPls concentration, which may reflect a higher uptake of NPls by the embryos/larvae at the highest tested concentrations. In addition, the increase of green fluorescence intensity appears to be directly related to the increase of NPls concentration, which was not altered on the co-exposures with SIM. A gradual green color intensity observed in some regions of the exposed embryos/larvae seems to be associated with the gradual NPls exposures. However, the autofluorescence of some structures of zebrafish embryos/larvae (e.g., yolk sac and eyes) in the same emission wavelength of the dragon green fluorophores (emission peak at 509 nm, we used the EGFP filter) complicated the image analysis because the autofluorescence may vary within and between embryos/larvae. The resulting interference did not allow an absolute/relative quantitative image analysis and then, to do a significant correlation with the intake of NPls.

Overall, this study shows that the NPls effects on bioavailability and toxicity of other contaminants cannot be ignored when we assess the environmental behavior and risks of plastic particles. In order to avoid the zebrafish autofluorescence “noise”, further studies should select, e.g., red colored NPls, to assess the NPls’ uptake, i.e., the visualization of NPls and autofluorescence cannot be made using the same filter (as it happened in the current study). More research studies should be performed to allow a better understanding of the biological processes involved on the effects of NPls with other contaminants. The assessment of endpoints, such as enzymatic and non-enzymatic antioxidant defenses, DNA and protein damage and expression of genes involved in metabolic pathways may be very useful to increase the knowledge about this issue.

## 5. Conclusions

The survival of the zebrafish embryos/larvae was affected by the combination of 1.5 mg/L NPls and SIM (12.5 or 15 µg/L) contrarily to single exposures of both contaminants. The highest NPls concentration tested in the combined exposures (1.5 mg/L) appears to be an effective and a greater carrier of SIM comparing to the lowest NPls concentration (0.015 mg/L). In terms of sub-lethal effects (hatching, heartbeat and malformations appearance), the effects of SIM were less pronounced or similar when in co-exposures with NPls, comparing with SIM single exposures. The results obtained from our study demonstrated that NPls may interact with SIM and modulate the SIM toxicity to zebrafish embryos/larvae and the effects of NPls on the toxicity of SIM was dependent on the NPls concentration. The resultant data highlights the importance of the environmental risk assessment when the contaminants are in mixtures and increase the concern about the possibility of NPls as a “vector” for other contaminants. Further studies assessing molecular and biochemical endpoints are very welcome, which will help to complement the results obtained in this study and to a better understanding about the mechanisms involved on the toxicity of NPls combined with other compounds.

## Figures and Tables

**Table 1:** Studies assessing the biological effects of polystyrene nanoplastics (PS NPls) to zebrafish (*Danio rerio*).

Size of PS NPls	Development Stage	Exposure Characteristics	Assessed Endpoints	Main Findings	Ref.
47 nm	Adult	Waterborne exposure 1 mg·L^−1^ 3 days Co-exposure: Bisphenol A (BPA)	Dopamine content Acetylcholinesterase (AChE) activity NPls quantification Gene/protein expression	NPls accumulated in various tissues. Inhibited AChE activity but not at the co-exposure. NPls caused myelin basic protein/gene up-regulation. Co-exposure increased the BPA uptake.	[3]
47 nm Microplastics (MPls; 41 µm)	Embryo	Waterborne exposure 1 mg·L^−1^ 120 hours (h) Co-exposure: 17 α-ethynylestradiol	Locomotor activity Body length Gene expression Antioxidant system AChE activity NPls quantification	NPls alone and co-exposure induced hypoactivity. Reduced the body larvae length. NPls caused gene upregulation. Decreased AChE activity and reduced glutathione content.	[1]
50, 200 and 500 nm	Embryo	Waterborne exposure 0.1 mg·mL^−1^ 6, 24 and 96 h Co-exposure: Chloroauric acid (HAuCl_4_)	Mortality Edemas Hatching Cell death Reactive oxygen species (ROS) Gene expression NPls accumulation	Smaller NPls readily penetrated the chorion and accumulated throughout the whole body. NPls induced only marginal effects, but the HAuCl_4_ synergistically exacerbated these effects in a dose and size dependent manner.	[5]
32 and 35 nm	Embryo and adult	Exposure via food 1 mg/fish gram 1 week	Reproduction Antioxidant system Mitochondrial function General physiology NPls distribution Locomotor activity	NPls modified the antioxidant system. Accumulated in the yolk sac. NPls transferred from mothers to offspring.	[4]
500 nm	Embryo	Waterborne exposure 1 mg·L^−1^ 48 h	NPls ingestion and tissue infiltration Protein carbonylation Antioxidant/detoxifying enzymes activities Swimming behavior	NPls infiltrated tissues. Decreased enzymatic activities. Altered the locomotor behaviour.	[11]
35 nm	Embryo	Waterborne exposure 0.1, 1 and 10 ppm 120 h	General physiology NPls uptake and distribution Locomotor activity Oxygen consumption	NPls accumulated in the yolk sac and migrated to other organs. Decreased the heartbeat rate and altered behavior.	[10]
27, 50, 217 and 727 nm	Embryo	Waterborne exposure 5, 25 and 50 mg·L^−1^ 48 h	Visualization of adsorbed, ingested or biodistributed NPls Eye width and length	The absorption was dependent on NPls size and time of exposure.	[14]
47 nm	Embryo	Waterborne exposure 0.1, 1 and 10 ppm 24, 48 and 96 h Co-exposure: Polycyclic aromatic hydrocarbons (PAHs)	Heartbeat rate Enzymatic activity Blood vessel formation Mitochondrial bioenergetics	NPls decreased the developmental deformities and impaired vascular development caused by PAHs. NPls decreased the mitochondrial coupling efficiency. NPls suggested sorbing PAHs and decreasing their uptake.	[15]
19 nm	Embryo	Waterborne exposure 0.2, 2 and 20 mg·L^−1^ 48 h	NPls distribution General physiology Cortisol and glucose levels Gene expression Larval behavior	NPls accumulated in various tissues. Affected swim bladder development. Increased cortisol and decreased glucose levels. NPls induced hyperactivity.	[13]

Ref., Reference. [3] Chen et al., [1] Chen et al., [5] Lee et al., [4] Pitt et al., [11] Parenti et al., [10] Pitt et al., [13] van Pomeren et al, [15] Trevisan et al., [14] Brun et al.

**Table 2:** At 0 and 96 h, hydrodynamic size and zeta potential average values of the polystyrene nanoplastics in the stock dispersion (at 0.001%) and in the medium used in the bioassays, at the tested concentrations (in the absence and presence of organisms and in combination with simvastatin (SIM)).

Nanoplastics	Hydrodynamic Size (nm)		Zeta Potential (mV)	
	0 h	96 h	0 h	96 h
Stock dispersion	69	69	−29	−29
In test media (without organisms)	66	66	−25	−25
In test media (with organisms)	68	294	−26	−23
In test media (with SIM)	77	305	−12	−12

**Table 3:** Median effect concentration (EC_50_) for zebrafish embryos/larvae exposed during 96 h to simvastatin (SIM) single versus combined with nanoplastics (NPls).

Endpoints	EC_50_ (µg/L)		
	SIM	SIM + 0.015 NPls	SIM + 1.5 NPls
Survival	15.39 ± 0.36	15.33 ± 25.07	6.93 ± 5.60
Malformations	9.71 ± 1.44	9.79 ± 2.09	n.d.
Hatching	13.96 ± 0.33	15.05 ± 8.86	n.d.
Heartbeat	19.71 ± 2.15	27.29 ± 7.13	n.d.

Results are presented as estimated value ± standard error. SIM + 0.015 NPls: 12.5 or 15 µg/L SIM + 0.015 mg/L NPls; SIM + 1.5 NPls: 12.5 or 15 µg/L SIM + 1.5 mg/L NPls; n.d.: Not determined. (The exposure condition induced 100% mortality, which did not allow to evaluate the other endpoints: malformations, hatching and heartbeat).

**Table 4:** Effects of single versus combined exposures of nanoplastics (NPls) and simvastatin (SIM) on mortality, hatching, heartbeat and malformations appearance on zebrafish embryos/larvae exposed for 96 h.

Time Exposure	Experimental Conditions							
	0.015 NPls	1.5 NPls	12.5 SIM	15 SIM	0.015 NPls + 12.5 SIM	0.015 NPls + 15 SIM	1.5 NPls + 12.5 SIM	1.5 NPls + 15 SIM
**Cumulative Mortality (%)**								
24 h	15.0 ± 5.0	15.0 ± 9.6	0.0 ± 0.0	2.5 ± 2.5	0.0 ± 0.0	0.0 ± 0.0	0.0 ± 0.0	10.0 ± 5.8
48 h	15.0 ± 5.0	20.0 ± 8.2	0.0 ± 0.0	25.0 ± 8.2	0.0 ± 0.0	20.0 ± 14.1	95.0 ± 5.0 *^#S^	95.0 ± 5.0 *
72 h	15.0 ± 5.0	20.0 ± 8.2	5.0 ± 5.0	32.5 ± 10.6	0.0 ± 0.0	20.0 ± 14.1	95.0 ± 5.0 *	95.0 ± 5.0 *
96 h	20.0 ± 8.2	20.0 ± 8.2	5.0 ± 5.0	40.0 ± 11.3	0.0 ± 0.0	25.0 ± 12.6	100.0 ± 0.0 *^#S^	100.0 ± 0.0 *
**Cumulative Hatching (%)**								
72 h	93.8 ± 6.3	83.3 ± 16.7	56.3 ± 16.3	34.2 ± 8.6 *	100.0 ± 0.0	77.5 ± 10.3	n.d.	n.d.
96 h	100.0 ± 0.0	91.7 ± 8.3	72.5 ± 11.1	51.7 ± 14.2 *	100.0 ± 0.0	100.0 ± 0.0 ^#S^	n.d.	n.d.
**Heartbeat (per minute)**								
48 h	131.0 ± 3.1	131.6 ± 0.8	98.2 ± 4.3 *	91.0 ± 2.4 *	122.6 ± 2.8	126.0 ± 1.8 ^#S^	n.d.	n.d.
**Cumulative Malformations (%)**								
48 h	6.3 ± 6.3	0.0 ± 0.0	40.0 ± 8.2	77.9 ± 10.9 *	70.0 ± 5.8 ^#N^	73.8 ± 12.5 *^#N^	n.d.	n.d.
72 h	6.3 ± 6.3	0.0 ± 0.0	63.8 ± 9.0 *	95.0 ± 5.0 *	75.0 ± 5.0 *^#N^	85.0 ± 9.6 *^#N^	n.d.	n.d.
96 h	6.3 ± 6.3	8.3 ± 8.3	80.0 ± 14.1 *	95.0 ± 5.0 *	75.0 ± 5.0 *^#N^	88.8 ± 6.6 *^#N^	n.d.	n.d.

Results are expressed in average value ± standard error. 0.015 NPls: 0.015 mg/L of nanoplastics; 1.5 NPls: 1.5 mg/L of nanoplastics; 12.5 SIM: 12.5 µg/L of simvastatin; 15 SIM: 15 µg/L of simvastatin; 0.015 NPls + 12.5 SIM: 0.015 mg/L of nanoplastics + 12.5 µg/L of simvastatin; 0.015 NPls + 15 SIM: 0.015 mg/L of nanoplastics + 15 µg/L of simvastatin; 1.5 NPls + 12.5 SIM: 1.5 mg/L of nanoplastics + 12.5 µg/L of simvastatin; 1.5 NPls + 15 SIM: 1.5 mg/L of nanoplastics + 15 µg/L of simvastatin; n.d.: Not determined (the exposure condition induced 100% mortality, which did not allow to evaluate the other endpoints, malformations, hatching and heartbeat). * Significant differences to control (*p* < 0.05). ^#S^ Significant differences to the correspondent single exposure of simvastatin (*p* < 0.05). ^#N^ Significant differences to the correspondent single exposure of nanoplastics (*p* < 0.05).

## Data Availability

The data are available on request from the corresponding author.

## Supplementary Materials

The following are available online at https://www.mdpi.com/2305-6304/9/3/44/s1, Table S1: Reference/calculated data of the selected functionalized polystyrene nanoplastics dispersion from the certificated sheet of the Bangs Laboratories, Inc.; Figure S1: Scanning electron microscopy (SEM) images of the functionalized polystyrene nanoplastics stock dispersion (at 0.001%); Figure S2: Schematic and microscopic visualization of embryo development of *Danio rerio* for 96 h, when exposed to control, solvent control, single and dual combinations of SIM and polystyrene NPls. Images resulted from the merge of two filters, bright field and enhanced green fluorescence protein (EGFP). SIM: simvastatin; NPls: nanoplastics; Single SIM exposures: 12.5 and 15 µg/L; Single NPls concentrations: 0.015 and 1.5 mg/L; Dual combinations: 0.015 mg/L NPls + 12.5 µg/L SIM; 0.015 mg/L NPls + 15 µg/L SIM; 1.5 mg/L NPls + 12.5 µg/L SIM; 1.5 mg/L NPls + 15 µg/L SIM.

## References

[1] Chen Q., Gundlach M., Yang S., Jiang J., Velki M., Yin D., Hollert H. (2017). Quantitative investigation of the mechanisms of microplastics and nanoplastics toward zebrafish larvae locomotor activity. Sci. Total Environ..

[2] Gigault J., Ter Halle A., Baudrimont M., Pascal P.-Y., Gauffre F., Phi T.-L., El Hadri H., Grassl B., Reynaud S. (2018). Current opinion: What is a nanoplastic?. Environ. Pollut..

[3] Chen Q., Yin D., Jia Y., Schiwy S., Legradi J., Yang S., Hollert H. (2017). Enhanced uptake of BPA in the presence of nanoplastics can lead to neurotoxic effects in adult zebrafish. Sci. Total Environ..

[4] Pitt J.A., Trevisan R., Massarsky A., Kozal J.S., Levin E.D., Di Giulio R.T. (2018). Maternal transfer of nanoplastics to offspring in zebrafish (*Danio rerio*): A case study with nanopolystyrene. Sci. Total Environ..

[5] Lee W.S., Cho H.-J., Kim E., Huh Y.H., Kim H.-J., Kim B., Kang T., Lee J.-S., Jeong J. (2019). Bioaccumulation of polystyrene nanoplastics and their effect on the toxicity of Au ions in zebrafish embryos. Nanoscale.

[6] Koelmans A.A., Besseling E., Shim W.J. (2015). Nanoplastics in the Aquatic Environment. Critical Review. Marine Anthropogenic Litter.

[7] (2019). Science Advice for Policy by European Academies a Scientific Perspective on Microplastics in Nature and Society. https://www.sapea.info/wp-content/uploads/report.pdf.

[8] Strungaru S.-A., Jijie R., Nicoara M., Plavan G., Faggio C. (2019). Micro- (nano) plastics in freshwater ecosystems: Abundance, toxicological impact and quantification methodology. TrAC Trends Anal. Chem..

[9] Brandts I., Teles M., Gonçalves A.P., Barreto A., Franco-Martinez L., Tvarijonaviciute A., Martins M.A., Soares A.M.V.M., Tort L., Oliveira M. (2018). Effects of nanoplastics on *Mytilus galloprovincialis* after individual and combined exposure with carbamazepine. Sci. Total Environ..

[10] Pitt J.A., Kozal J.S., Jayasundara N., Massarsky A., Trevisan R., Geitner N., Wiesner M., Levin E.D., Di Giulio R.T. (2018). Uptake, tissue distribution, and toxicity of polystyrene nanoparticles in developing zebrafish (*Danio rerio*). Aquat. Toxicol..

[11] Parenti C.C., Ghilardi A., Della Torre C., Magni S., Del Giacco L., Binelli A. (2019). Evaluation of the infiltration of polystyrene nanobeads in zebrafish embryo tissues after short-term exposure and the related biochemical and behavioural effects. Environ. Pollut..

[12] Kashiwada S. (2006). Distribution of nanoparticles in the see-through medaka (*Oryzias latipes*). Environ. Health Perspect..

[13] Van Pomeren M., Brun N.R., Peijnenburg W.J.G.M., Vijver M.G. (2017). Exploring uptake and biodistribution of polystyrene (nano)particles in zebrafish embryos at different developmental stages. Aquat. Toxicol..

[14] Brun N.R., van Hage P., Hunting E.R., Haramis A.P.G., Vink S.C., Vijver M.G., Schaaf M.J.M., Tudorache C. (2019). Polystyrene nanoplastics disrupt glucose metabolism and cortisol levels with a possible link to behavioural changes in larval zebrafish. Commun. Biol..

[15] Trevisan R., Voy C., Chen S., Di Giulio R.T. (2019). Nanoplastics Decrease the Toxicity of a Complex PAH Mixture but Impair Mitochondrial Energy Production in Developing Zebrafish. Environ. Sci. Technol..

[16] Ribeiro S., Torres T., Martins R., Santos M.M. (2015). Toxicity screening of diclofenac, propranolol, sertraline and simvastatin using *Danio rerio* and paracentrotus lividus embryo bioassays. Ecotoxicol. Environ. Saf..

[17] Cunha V., Santos M.M., Moradas-Ferreira P., Castro L.F.C., Ferreira M. (2017). Simvastatin modulates gene expression of key receptors in zebrafish embryos. J. Toxicol. Environ. Health Part A Curr. Issues.

[18] Dong Z., Senn D.B., Moran R.E., Shine J.P. (2013). Prioritizing environmental risk of prescription pharmaceuticals. Regul. Toxicol. Pharmacol..

[19] Pereira A.M.P.T., Silva L.J.G., Meisel L.M., Lino C.M., Pena A. (2015). Environmental impact of pharmaceuticals from Portuguese wastewaters: Geographical and seasonal occurrence, removal and risk assessment. Environ. Res..

[20] Pereira A.M.P.T., Silva L.J.G., Laranjeiro C.S.M., Meisel L.M., Lino C.M., Pena A. (2017). Human pharmaceuticals in Portuguese rivers: The impact of water scarcity in the environmental risk. Sci. Total Environ..

[21] Almeida M., Martins M.A., Soares A.M.V., Cuesta A., Oliveira M. (2019). Polystyrene nanoplastics alter the cytotoxicity of human pharmaceuticals on marine fish cell lines. Environ. Toxicol. Pharmacol..

[22] Neuparth T., Martins C., Carmen B., Costa M.H., Martins I., Costa P.M., Santos M.M. (2014). Hypocholesterolaemic pharmaceutical simvastatin disrupts reproduction and population growth of the amphipod *Gammarus locusta* at the ng/L range. Aquat. Toxicol..

[23] Barros S., Montes R., Quintana J.B., Rodil R., André A., Capitão A., Soares J., Santos M.M., Neuparth T. (2018). Chronic environmentally relevant levels of simvastatin disrupt embryonic development, biochemical and molecular responses in zebrafish (*Danio rerio*). Aquat. Toxicol..

[24] Patibandla S., Jiang J.-Q., Shu X. (2018). Toxicity assessment of four pharmaceuticals in aquatic environment before and after ferrate (VI) treatment. J. Environ. Chem. Eng..

[25] Liu Y., Ding R., Pan B., Wang L., Liu S., Nie X. (2019). Simvastatin affect the expression of detoxification-related genes and enzymes in *Daphnia magna* and alter its life history parameters. Ecotoxicol. Environ. Saf..

[26] Campos L.M., Rios E.A., Guapyassu L., Midlej V., Atella G.C., Herculano-Houzel S., Benchimol M., Mermelstein C., Costa M.L. (2016). Alterations in zebrafish development induced by simvastatin: Comprehensive morphological and physiological study, focusing on muscle. Exp. Biol. Med..

[27] Wang C., Ku P., Nie X., Bao S., Wang Z., Li K. (2019). Effects of simvastatin on the PXR signaling pathway and the liver histology in Mugilogobius abei. Sci. Total Environ..

[28] Campos L.M., Rios E.A., Midlej V., Atella G.C., Herculano-Houzel S., Benchimol M., Mermelstein C., Costa M.L. (2015). Structural Analysis of Alterations in Zebrafish Muscle Differentiation Induced by Simvastatin and Their Recovery with Cholesterol. J. Histochem. Cytochem..

[29] (2013). OECD Test Number 210: Fish, Early-Life Stage Toxicity Test.

[30] Al-Sid-Cheikh M., Rowland S.J., Stevenson K., Rouleau C., Henry T.B., Thompson R.C. (2018). Uptake, Whole-Body Distribution, and Depuration of Nanoplastics by the Scallop *Pecten maximus* at Environmentally Realistic Concentrations. Environ. Sci. Technol..

[31] (2019). OECD Guidance Document on Aquatic Toxicity Testing of Difficult Substances and Mixtures.

[32] Walkey C.D., Chan W.C.W. (2012). Understanding and controlling the interaction of nanomaterials with proteins in a physiological environment. Chem. Soc. Rev..

[33] Galloway T.S., Cole M., Lewis C. (2017). Interactions of microplastic debris throughout the marine ecosystem. Nat. Ecol. Evol..

[34] Lee H., Shim W.J., Kwon J.H. (2014). Sorption capacity of plastic debris for hydrophobic organic chemicals. Sci. Total Environ..

[35] Wang J., Tan Z., Peng J., Qiu Q., Li M. (2016). The behaviors of microplastics in the marine environment. Mar. Environ. Res..

