# Supplementary material for: Polystyrene Nanoplastics Can Alter the Toxicological Effects of Simvastatin on Danio rerio
Source: Toxics. 2021 Feb 26;9(3):44. doi: 10.3390/toxics9030044 (PMC7996764; doi:10.3390/toxics9030044)
Supplement: Supplementary file 1 [file toxics-09-00044-s001.pdf]

Article

Supplementary Material: Polystyrene Nanoplastics Can Alter the Toxicological Effects of Simvastatin on *Danio rerio*

Angela Barreto, Joana Santos, Mónica J.B. Amorim and Vera L. Maria

S1. Materials and Methods

Table S1. Reference/calculated data of the selected functionalized polystyrene nanoplastics dispersion from the certificated sheet of the Bangs Laboratories, Inc.

Calculated Data	Values
Density (g/cm^3)	1.06
Number of nanospheres/g	8.341×10^{15}
Number of nanospheres/ml	8.346×10^{13}
Surface area (μm^2/g)	9.434×10^{13}
Solids content (%)	1

The NPls' uptake traceability characterization was made using embryos (n = 12; 3 biological replicates per condition and per time of exposure) exposed to the same conditions (temperature, test duration, exposure concentrations and volume of test medium/embryo) used in the FET test design in a parallel additional experiment. However, the test ran in the dark (the glass Petri dishes were covered with aluminum foil to avoid contact with light and consequent fluorescence loss). The samples were collected at 24, 48, 72 and 96 h, under a stereomicroscope. The embryos/larvae were washed with distilled water, anesthetized with 4% tricaine (E10521, Sigma-Aldrich, Saint Louis, MO, USA) and mounted onto concave microscope slides with tricaine solution (4%), prior to observation with a fluorescence microscope (Axio Imager Z19, with AxioCam HR, Zeiss, White Plains, NY, USA). The filter used to analyze the green fluorescence was EGFP (excitation 450–490 nm, emission 500–550 nm).

2. Results

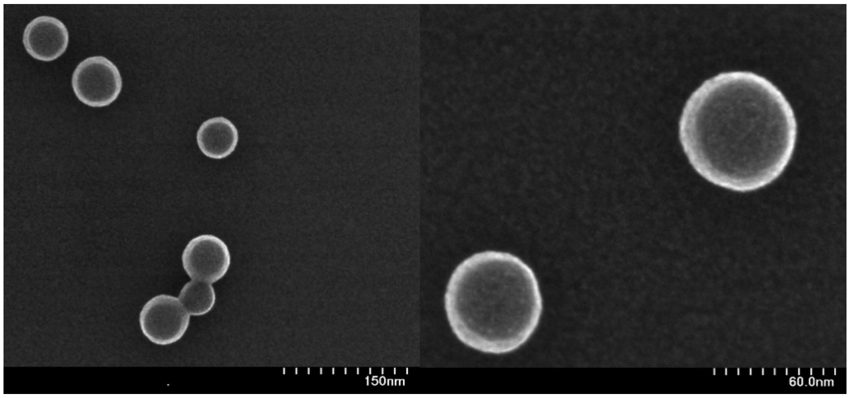

Figure S1. Scanning electron microscopy (SEM) images of the functionalized polystyrene nanoplastics stock dispersion (at 0.001%).

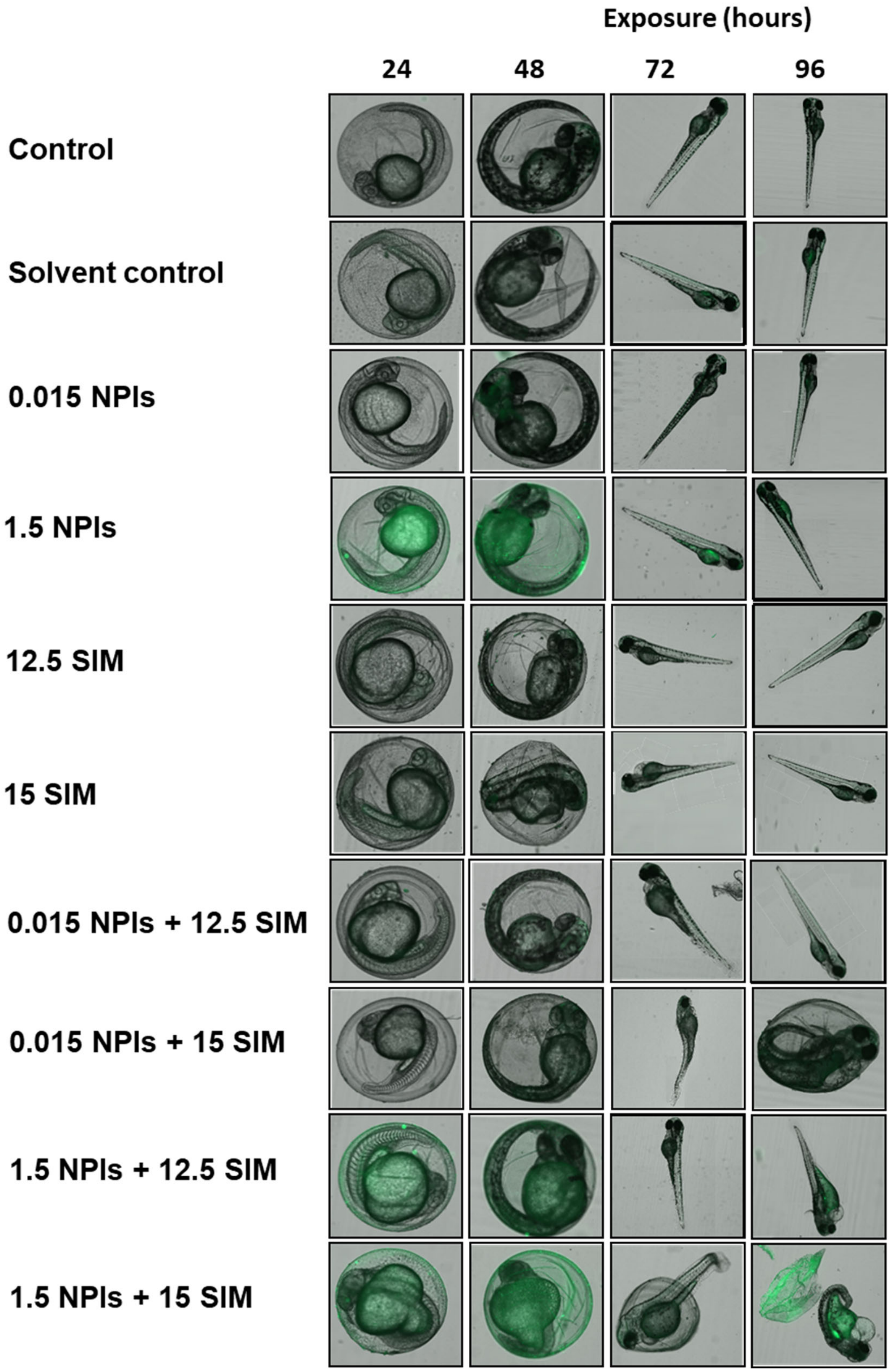

Figure S2. Schematic and microscopic visualization of embryo development of *Danio rerio* for 96 hours, when exposed to control, solvent control, single and dual combinations of simvastatin (SIM) and polystyrene nanoplastics (NPls). Images resulted from the merge of two filters, brightfield and enhanced green fluorescence protein (EGFP). SIM: simvastatin; NPls: nanoplastics; Single SIM exposures: 12.5 and 15 μg/L; Single NPls concentrations: 0.015 and 1.5 mg/L; Dual combinations: 0.015 mg/L NPls + 12.5 μg/L SIM; 0.015 mg/L NPls + 15 μg/L SIM; 1.5 mg/L NPls + 12.5 μg/L SIM; 1.5 mg/L NPls + 15 μg/L SIM.
